# Identification of significant precursor gases of secondary organic aerosols from residential wood combustion

**DOI:** 10.1038/srep27881

**Published:** 2016-06-17

**Authors:** Emily A. Bruns, Imad El Haddad, Jay G. Slowik, Dogushan Kilic, Felix Klein, Urs Baltensperger, André S. H. Prévôt

**Affiliations:** 1 Laboratory of Atmospheric Chemistry, Paul Scherrer Institute, 5232 Villigen, Switzerland

## Abstract

Organic gases undergoing conversion to form secondary organic aerosol (SOA) during atmospheric aging are largely unidentified, particularly in regions influenced by anthropogenic emissions. SOA dominates the atmospheric organic aerosol burden and this knowledge gap contributes to uncertainties in aerosol effects on climate and human health. Here we characterize primary and aged emissions from residential wood combustion using high resolution mass spectrometry to identify SOA precursors. We determine that SOA precursors traditionally included in models account for only ~3–27% of the observed SOA, whereas for the first time we explain ~84–116% of the SOA by inclusion of non-traditional precursors. Although hundreds of organic gases are emitted during wood combustion, SOA is dominated by the aging products of only 22 compounds. In some cases, oxidation products of phenol, naphthalene and benzene alone comprise up to ~80% of the observed SOA. Identifying the main precursors responsible for SOA formation enables improved model parameterizations and SOA mitigation strategies in regions impacted by residential wood combustion, more productive targets for ambient monitoring programs and future laboratories studies, and links between direct emissions and SOA impacts on climate and health in these regions.

Atmospheric aerosols have multifaceted, yet poorly understood, effects on climate^1^, human health^2^ and visibility^3^. Organic aerosol (OA) is a large fraction of the total atmospheric submicron aerosol burden^4^ and consists of primary organic aerosol (POA) emitted directly into the atmosphere and secondary organic aerosol (SOA) formed from the condensation of oxidation products with sufficiently low volatility^5^. SOA mass loadings often exceed those of POA^4^ and while uncertainties remain in POA characterization, SOA formation and evolution in the atmosphere are even less understood^5^. For example, the most recent estimates of global SOA production show order of magnitude differences between “top-down” approaches based on ambient measurements and “bottom-up” approaches based on models using emission inventories and SOA yields determined from laboratory smog chamber experiments^5^,^6^,^7^,^8^. Gaps between measurements and models have been reduced by the recent implementation of the volatility basis set approach^8^,^9^,^10^,^11^,^12^,^13^,^14^,^15^,^16^,^17^, where emissions are assumed to contain significant amounts of semi-volatile and intermediate volatility organic compounds (S/IVOCs). S/IVOC aging products can contribute to SOA in addition to aging products from traditional higher volatility precursors such as benzene, toluene and α-pinene. However, the contributions of individual S/IVOCs to the total carbon balance are largely unmeasured, which severely impedes the representation of SOA chemical composition, yields and production rates in models and thus understanding of climate and health effects.

Residential wood combustion can contribute considerably to the atmospheric OA burden^18^,^19^,^20^, particularly in regions with cooler climates, through both primary emissions and significant SOA formation^21^,^22^. Previous work shows that traditional SOA precursors account for less than 20% of the observed SOA formed from residential wood combustion emissions and a significant fraction of the SOA is hypothesized to derive from S/IVOCs^22^. However, few studies have measured S/IVOCs from residential wood combustion^23^,^24^,^25^,^26^,^27^,^28^ and the evolution of these S/IVOCs with aging (including the contribution to SOA formation) is unknown. Here, we identify the main species responsible for SOA formation during the aging of residential wood combustion emissions generated during stable burning conditions using a high resolution proton transfer reaction time-of-flight mass spectrometer (PTR-ToF-MS) and a high resolution time-of-flight aerosol mass spectrometer (AMS). While this study quantitatively explains the majority of SOA formation from any complex anthropogenic emission source for the first time and provides a critical and substantial leap forward in our understanding of SOA formation, only a narrow range of residential burning conditions were investigated. However, the work presented here provides a clear path forward for investigating SOA formation under different burning conditions and from other sources.

## Results and Discussion

### Contribution of individual gas-phase organics to observed SOA

Emissions from a residential wood burner are injected into a smog chamber, where atmospheric aging is simulated (see Methods). POA, equivalent black carbon (eBC) and non-methane organic gases (NMOGs) are directly emitted during each burn and emission factors are in agreement with recently published results generated under similar burning conditions (see Table 1)^23^,^29^,^30^,^31^. Aging of the emissions yields 3 to 7 times more SOA than directly emitted OA after reaching a hydroxyl radical (OH) exposure of (4.5–5.5) × 10^7^ molec cm^−3^ h (see Table 1 and Supplementary Fig. S1), which corresponds to ~2 days of aging in the atmosphere at an OH concentration of 1 × 10^6^ molec cm^−3^. The PTR-ToF-MS monitors NMOGs with a proton affinity greater than that of water and while high resolution mass spectra allow assignment of molecular formulas to observed ions, structural information is not retrieved. However, previous studies focused on complete characterization of residential wood combustion emissions have identified over 85% of the measured NMOG mass^26^,^28^, which provides direction for assigning structures to the molecular formulas identified using the PTR-ToF-MS. Approximately 70% of the NMOG mass measured using the PTR-ToF-MS is structurally assigned based on previously identified residential wood combustion products^23^,^24^,^25^,^26^,^27^,^28^,^32^.

**Figure 1:**
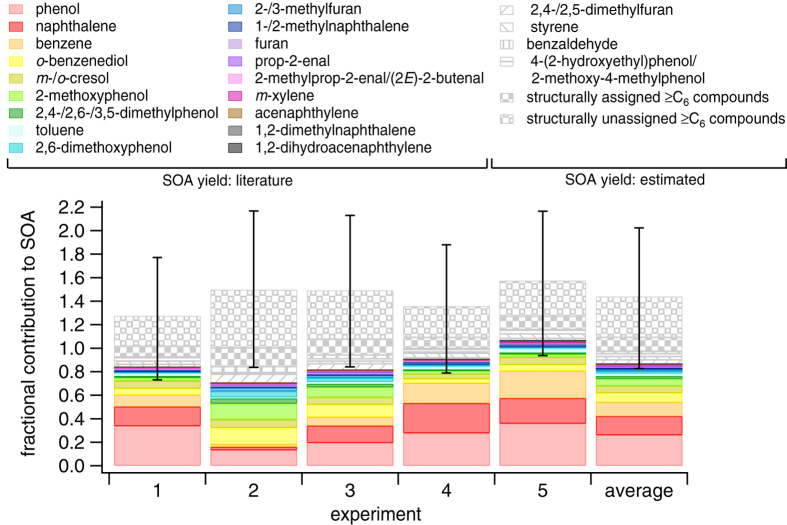
Fractional contribution of 22 individual NMOGs and two lumped NMOG categories to observed SOA for each experiment and the average of all experiments. Contributions are determined after exposure to (4.5–5.5) × 10^7^ molec cm^−3^ h OH. Solid bars represent individual species for which SOA yields are published and patterned bars represent species for which SOA yields are estimated. Error bars correspond to the range of possible contributions assuming the lowest and highest reasonable SOA yields for each compound. The yield uncertainty is estimated to be ±50% for species with estimated SOA yields and for *o*-benzenediol as only a single measurement is available in the literature.

NMOGs undergo oxidation during atmospheric aging to form a variety of products, some of which remain in the gas phase and others which have sufficiently low volatility to partition to the particle phase. The contribution of an individual NMOG to SOA is the product of the amount reacted at a certain OH exposure and the best estimate SOA yield determined from the literature (see Supplementary Information for discussion of applied yields, Table S1). Eighteen of the identified NMOGs have published SOA yields (phenol, naphthalene, benzene, *o*-benzenediol, isomers of *m*-/*o*-cresol, 2-methoxyphenol, isomers of 2,4-/2,6-/3,5-dimethylphenol, toluene, 2,6-dimethoxyphenol, isomers of 2-/3-methylfuran, isomers of 1-/2-methylnaphthalene, furan, prop-2-enal, isomers of 2-methylprop-2-enal/(2*E*)-2-butenal, *m*-xylene, acenaphthylene, 1,2-dimethylnaphthalene and 1,2-dihydroacenaphthylene)^33^,^34^,^35^,^36^,^37^,^38^,^39^,^40^,^41^. For compounds expected to contribute to SOA formation based on structure, but for which SOA yields are not available in the literature, the yield is estimated as the average of the published SOA yields applied to the NMOGs with at least six carbon atoms per molecule (≥C_6_). Using this estimated SOA yield, four additional ≥C_6_ compounds (isomers of 2,4-/2,5-dimethylfuran, styrene, benzaldehyde and isomers of 4-(2-hydroxyethyl)phenol/2-methoxy-4-methylphenol) are considered individually as each contributes at least 3% to the observed SOA in at least one experiment. Figure 1 shows the contribution of these 22 individual NMOGs to the observed SOA for five individual burns and the average of these burns (see Table 2 for values in Fig. 1). In all experiments, 84-116% of the observed SOA is accounted for by these 22 NMOGs, which is considerably more than the <20% explained previously only considering SOA formed from traditional precursors^22^. SOA yields depend on factors such as the presence of seed aerosol, the ratio of NO_x_ (NO+NO_2_) to NMOG, and OA mass loadings. For example, the ratio of NO_x_/NMOG can impact yields by shifting the volatility distribution of oxidation products, although the direction of this change is NMOG-dependent and in some cases, changes in NO_x_/NMOG have no impact on measured yields. Applied yields are selected from experiments conducted under the most similar conditions as the current study. Error bars in Fig. 1 correspond to the range of possible contributions using upper and lower limits of the best estimate SOA yields (see Supplementary Table S1). Even applying the lowest reasonable SOA yields, at least 50% of the observed SOA is explained by the oxidation of these 22 NMOGs.

Figure 1 clearly indicates that the most important SOA precursors present in the wood smoke emitted during stable residential burning of beech wood include not only traditional compounds such as benzene and alkyl-benzenes, but also phenols, naphthalene and alkyl-naphthalenes. The non-traditional compounds contributing to SOA formation are highly reactive (lifetimes of <1 d at 1 × 10^6^ molec cm^−3^ OH and 294–298 K) and efficient SOA precursors and while their oxidation products contribute over 70% to the observed SOA, they are not traditionally considered in transport models. The reaction rate constants with OH of the majority of these compounds have weak temperature dependence^42^ suggesting that even at lower temperatures, when residential wood combustion is most prevalent, SOA formation is substantial and occurs rapidly^43^.

Wood combustion produces highly variable emission profiles depending on parameters such as wood type, combustion conditions and appliance type^21^,^22^,^23^,^24^,^25^,^26^,^27^,^28^,^32^. Interestingly, although emission profiles vary between burns resulting in different contributions of individual NMOGs to SOA, in all cases these 22 NMOGs explain the large majority of observed SOA. For example, phenol, benzene and naphthalene contribute only ~20% to the observed SOA in experiment 2, compared to ~80% in experiment 5, whereas 2-methoxyphenol, 2,6-dimethoxyphenol, and *o*-benzenediol contribute ~33% to the observed SOA in experiment 2, compared to only ~8% in experiment 5, indicating that the finding that few NMOGs contribute to the majority of SOA formation is not limited to a narrow range of wood combustion emission profiles.

SOA yields in the literature are limited to a small fraction of the hundreds to thousands of NMOGs in the atmosphere. In addition to the 22 NMOGs, additional species possibly contributing to SOA for which yields are unknown are accounted for by lumping into two categories: compounds that have been assigned a structure based on previous wood combustion experiments^23^,^24^,^25^,^26^,^27^,^28^,^32^ and have at least six carbon atoms per molecule (structurally assigned ≥C_6_ compounds, listed individually in Table S2) and compounds for which no structures have been assigned, but are expected to have at least six carbon atoms per molecule due to the high molecular weight (structurally unassigned ≥C_6_ compounds). For both lumped categories, the SOA yield is estimated as the average of the published yields applied to the NMOGs with at least six carbon atoms per molecule. The contributions to SOA from these two additional categories are shown in Fig. 1 and when combined with the 22 individual NMOGs, explain a total of ~128–157% of the observed SOA, which although greater than 100%, is within the uncertainties considering the assumptions of the approach.

### Linking NMOG and SOA composition

**Figure 2:**
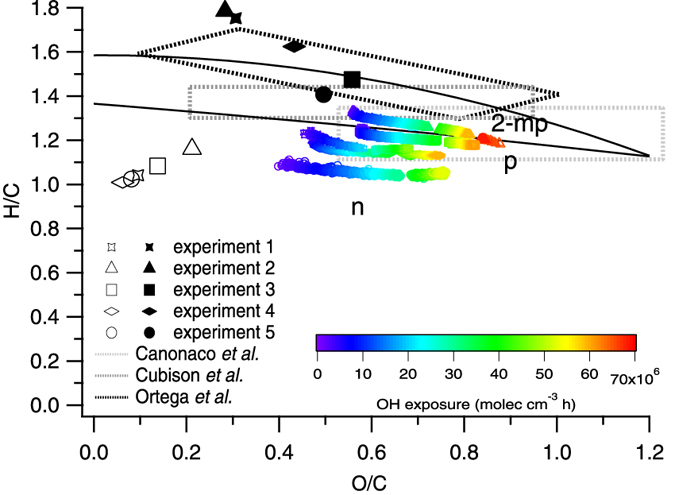
Elemental composition of SOA, POA and mass concentration-weighted average of 22 primary NMOGs. Colored traces correspond to SOA as a function of OH exposure, open black data points correspond to NMOGs and solid black data points correspond to POA for each experiment. Lettered data points correspond to literature values of SOA formed during aging of individual precursors (phenol, p; naphthalene, n; 2-methoxyphenol, 2-mp)^34^,^47^. Solid black lines correspond to the region encompassing typical ambient experiments^46^ and dashed gray lines encompass measurements of laboratory SOA from open biomass burning^48^ and ambient OA measurements impacted by open biomass burning^49^ and residential burning^50^.

SOA composition provides further insight into the NMOGs responsible for SOA formation. Figure 2 shows the SOA elemental composition when SOA/POA ≥1 during all five experiments determined from AMS data using the Aiken parameterizations^44^ (see Supplementary Figs S2 and S3 for further chemical analysis of the total OA and SOA using traditional and new AMS elemental analysis parameterizations)^44^,^45^. Aiken parameterizations are used to facilitate comparison with previous studies. Relative to typical ambient measurements (region encompassed by solid black lines)^46^, SOA formed during these experiments is located near the lower limits of H/C observed for a given O/C. These SOA H/C values are comparable to and correlate with those of the mass concentration-weighted average of the 22 primary NMOGs discussed above (open black data points). For example, the SOA and NMOG H/C ratios are both highest in experiments 2 and 3. The similarities of the chemical features of the SOA (e.g., H/C) and the NMOGs provide further evidence that these precursors are responsible for the observed SOA. The end point of published oxidation experiments of individual NMOGs which contribute at least 10% to the total observed SOA in the current experiments are also included in Fig. 2. The end points of the current experiments are similar in terms of H/C to published oxidation experiments of phenol^34^. Similar agreement in endpoints is observed with SOA formed from the oxidation of 2-methoxyphenol^34^, with the best agreement for experiment 2, which has the highest relative contribution to the observed SOA from this compound. Naphthalene SOA^47^ has a lower H/C than the SOA in the current experiments, as well as the bulk primary NMOG values, however the SOA is an average of all contributing NMOGs, most of which have a larger H/C than naphthalene.

Laboratory measurements of aged open biomass burning emissions^48^ and ambient measurements of air masses influenced by open biomass burning^49^ and residential wood combustion^50^ are also shown in Fig. 2. Elemental ratios from literature data are determined using the parameterizations of Ng *et al*.^46^ where applicable. Ratios of H/C from the current experiments are within the range of the ambient open and residential burning, although the range is large and encompasses a range of degrees of oxidation, indicating these findings are atmospherically relevant and may be applicable to a range of residential burning conditions as well as open burning.

### New directions for monitoring, mitigation, modelling and experimental programs

The finding that relatively few NMOGs contribute to the majority of SOA from residential wood combustion under the studied conditions, as well as the identification of these NMOGs, has implications for routine ambient measurement strategies, SOA mitigation strategies, model parameterizations, directions for future laboratory studies and investigations into SOA composition and effects on health.

The results here indicate that routine monitoring of NMOGs forming SOA from residential wood combustion is possible due to the relatively low number of contributing species. Currently, the majority of the 22 NMOGs are not typically monitored routinely in the atmosphere, resulting in a critical gap in emission inventories^51^ that needs to be remedied to improve modelling efforts of SOA. Measurement of these NMOGs during future emission studies is also critical to determine whether these compounds can explain SOA formation from emissions generated during different burning conditions and from other sources; as described below, calculations suggest that these NMOGs likely also contribute considerably to open biomass burning SOA. The methodology applied here can be applied to other emission sources, including open biomass burning and fossil fuel combustion. Mitigation strategies, such as the implementation of catalytic converters, to reduce the emissions of NMOGs which on average contribute the most to SOA formation can greatly reduce SOA formation from residential wood combustion.

On average, the three largest contributors to SOA formation are phenol, naphthalene and benzene, which together contribute up to ~80% of the total observed SOA, however phenol and naphthalene are not among the traditional SOA precursors typically included in models. In these experiments, traditional SOA precursors above the instrument detection limit (i.e., benzene, toluene and *m*-xylene) account for only 3–27% of the observed SOA, which is in agreement with previous findings^22^. The under-prediction of SOA from traditional precursors can be particularly severe depending on the emission profile. For example, traditional SOA precursors contribute only 3% to the observed SOA in experiment 2, highlighting the importance of non-traditional SOA precursors in the formation of residential wood combustion SOA. Identification of the individual NMOGs contributing to SOA formation provides models with the insight necessary to transition from using lumped categories to represent S/IVOCs contributing to SOA formation to more explicit approaches, although implementation of more explicit SOA formation into the volatility basis set framework requires future work. While below the detection limit in the current experiments, monoterpene emissions can be significant under different burning conditions and are known to contribute to SOA formation and should thus also be considered in the modelling of residential wood combustion SOA (see Supplementary Information for further discussion of monoterpenes). An important parameter required in models is the ratio between non-traditional NMOGs and semi-volatile POA components, estimated to be 4 ± 1, based on our OA and PTR-ToF-MS measurements and the volatility distribution function of May *et al*.^52^.

**Figure 3:**
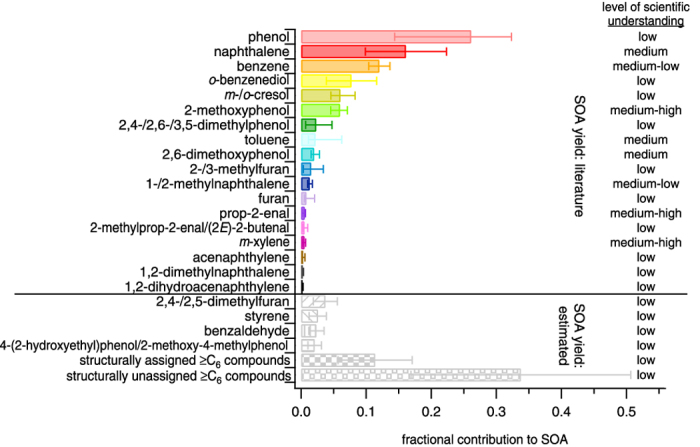
Average fractional contribution to observed SOA and level of scientific understanding of 22 individual NMOGs and two lumped NMOG categories. Contributions are determined after exposure to (4.5–5.5) × 10^7^ molec cm^−3^ h OH. Solid bars represent individual species for which SOA yields are published and patterned bars represent species for which SOA yields are estimated. Error bars correspond to the range of possible contributions assuming the lowest and highest reasonable SOA yields for each compound. The yield uncertainty is estimated to be ±50% for species with estimated SOA yields and for *o*-benzenediol as only a single measurement is available in the literature. The level of scientific understanding is based on the number of studies reporting SOA yields, agreement between studies, and similarity to experimental conditions in the current study.

Figure 3 details the average contribution of the 22 individual NMOGs and two additional lumped categories to SOA. A level of scientific understanding is assigned to each species based on published SOA yield data including the number of studies, agreement between studies and similarity to experimental conditions in the current study. The level of scientific understanding combined with the relative contribution to the observed SOA identifies knowledge gaps in which future research should focus to constrain the data needed to understand SOA formation. For example, phenol is one of the top three contributors to SOA in all experiments, however, the scientific understanding is low as only one paper has published SOA yields for this compound with seed aerosol^37^ and the experiments were conducted with NO_x_/NMOG ratios of 0 or ~5600–8300 ppb ppmC^−1^, whereas the current experiments are conducted at NO_x_/NMOG ratios of ~35–350 ppb ppmC^−1^. While the NO_x_/NMOG ratios for the current experiments are dissimilar to those under which yields were measured, the ratios fall within the large literature range and thus, uncertainties represented in Figs 1 and 3 are expected to encompass the actual value. In addition to individual NMOGs, pre-defined NMOG mixtures could be tested to determine combined yields simulating more complex mixtures.

### Implications for optical properties and health

Recent studies indicate that biomass burning SOA absorbs more strongly in the short-visible and near-UV range and less strongly in the long-range visible range in comparison to POA^53^. An explanation for this trend is provided by the current study, in which 17 of the 22 NMOGs discussed above have at least one aromatic ring. The SOA from these aromatic compounds likely retains some of their absorptive features (e.g., conjugated double bonds). Compounds with more rings are expected to have lower volatility and increasingly partition to POA, whereas the main contributors to SOA are relatively small aromatics, which do not partition to the particle phase until oxidized. An increase in small aromatics in the OA with aging would shift the OA light absorbing properties towards short wavelengths, because as the number of fused rings decreases, absorption shifts to shorter wavelengths. The identification of NMOGs participating in SOA formation improves the understanding of SOA composition and properties, which are critical to deduce effects on climate and health.

Although the majority of the observed SOA mass is accounted for by oxidation of 22 NMOGs, SOA mass and toxicity are not necessarily correlated and further study is needed to deduce the impacts of residential wood combustion SOA on health. However, aromatic compounds, which contribute to the majority of observed SOA, and their functionalized analogs are known to have particularly deleterious health effects^54^. The identification of the NMOGs contributing to SOA from residential wood combustion provides direction for future health studies.

### Implications for open biomass burning SOA

Residential wood combustion is known to dominate local and regional OA under some conditions^18^,^19^, for example during winter in Europe, but biomass burning OA is likely dominated by open fires rather than residential wood combustion on a global scale. The relative emission factors of the NMOGs in open biomass burning can be very different to those in residential wood burning, however, recent work estimating the SOA potential from open biomass burning emissions also suggests that non-traditional species, such as phenols, benzenediols and benzaldehyde, dominate SOA production^55^. To date, a comprehensive analysis of the NMOG-SOA closure has not been performed for open biomass burning. However, emission factors for 19 of the 22 NMOGs investigated here have been reported for a variety of open burns (exceptions are acenaphthylene, 1,2-dihydroacenaphthylene and 1,2-dimethylnaphthalene)^56^. Given these inventories, the estimated SOA yields (see Table 2), and the total biomass burned annually^57^, these 19 NMOGs contribute ~4 TgC yr^−1^ (estimated range of 0.4–20 TgC yr^−1^ based on range of emission factors and best estimate SOA yields) to global biomass burning SOA, which considering the uncertainties in this approach, is within the range of the best top-down estimate of 17 TgC yr^−1^ SOA (estimated range of 0–34 TgC yr^−1^) (Table 3)^5^. Although this is a very uncertain calculation and the mass closure is unsurprisingly not as good as that for the residential wood combustion system, the results suggest that the NMOGs identified above are possibly critical targets for monitoring and mitigation initiatives with respect to global biomass burning, although more work is needed to determine if additional NMOGs contribute to open biomass burning SOA.

Although additional work is needed to definitively establish the dominant NMOG precursors to SOA across all fuel types and burn conditions, the present study indicates that a limited number of non-traditional precursors may be dominant factors in the global SOA budget. These critical NMOGs are not typically included in long-term monitoring initiatives and the level of scientific understanding of their SOA production is generally low. Nonetheless there is evidence that SOA from these NMOGs contributes significantly to detrimental health and climate-related properties, making these NMOGs of high priority for future experimental and mitigation efforts.

## Methods

Beech wood (*Fagus sylvatica*) is combusted in a modern woodstove (Avant, 2009, Attika) and the emissions are sampled from the chimney through a heated line (473 K), diluted using an ejector diluter (473 K, DI-1000, Dekati Ltd.) and injected into a Teflon smog chamber (~7 m^3^, 287 K, 55% relative humidity) through a heated line (423 K). Non-refractory primary particulate emissions are characterized using a high resolution time-of-flight aerosol mass spectrometer (AMS, 1 μm lens, 600 °C vaporizer temperature, Aerodyne Research, Inc.). NMOGs are sampled through a heated line (323 K) and characterized using a high resolution proton transfer reaction time-of-flight mass spectrometer (PTR-ToF-MS 8000, H_3_O^+^ reagent ion, Ionicon Analytik G.m.b.H.; see Supplementary Table S3 for reagent ion reaction rates). After characterizing the primary emissions, a single injection of d9-butanol (butanol-D9, 98%, Cambridge Isotope Laboratories) and a continuous injection of nitrous acid in pure air (2.3–2.6 l min^−1^, ≥99.999%, Air Liquide) are introduced into the chamber and the contents of the chamber are irradiated with UV light (40 lights, 90–100 W, Cleo Performance, Philips) for 4–6.5 h to simulate atmospheric aging. The evolution of the gas-phase and particulate phase composition and concentration are monitored in real-time throughout aging. The mass of reacted NMOGs throughout the experiment is measured and the corresponding contribution to SOA is determined using SOA mass yields reported in the literature^33^,^34^,^35^,^36^,^37^,^38^,^39^,^40^,^41^. Mass loading dependent yields are not available for all species, but when available, the end points are taken as they are representative of the current experiments based on OA mass loadings (Table 1, Fig. S1). SOA is calculated as the difference between total OA and POA. Separation of SOA and POA components in total OA mass spectra was attempted using positive matrix factorization (PMF), which employs a linear combination of static factor profiles and corresponding time-dependent intensities to represent a mass spectral time series. PMF was implemented using the multilinear engine (ME-2), with model configuration and data analysis performed with the source finder toolkit (SoFi). Improved factor separation is possible with ME-2 compared to conventional PMF analysis due to the full exploration of the rotational ambiguity of the solution space. However, the POA factor was relatively unstable, with small changes in the mass spectra leading to large differences in the time series. The uncertainties in the PMF analysis were mostly in the POA fraction. Therefore, the only alternative was to calculate SOA as the difference between total OA and POA. The large SOA/POA ratios (~3–7) in these experiments indicate that even if some POA is replaced with SOA, this would have a small impact on the SOA concentration. All values corresponding to aged emissions are taken at an OH exposure of (4.5–5.5) × 10^7^ molec cm^−3^ h (exact OH exposures for each experiment are given in Table 1). Full details of the experiments and data analysis are located in the Supplementary Information.

## Additional Information

**How to cite this article**: Bruns, E. A. *et al*. Identification of significant precursor gases of secondary organic aerosols from residential wood combustion. *Sci. Rep.* **6**, 27881; doi: 10.1038/srep27881 (2016).

## Supplementary Material

Supplementary Information

## Figures and Tables

**Table 1:** Characteristics of primary and aged products in the smog chamber^a^.

experiment	wood burned perchamber volume(g m^−3^)	CO_2_(ppm)	CO(ppm)	CH_4_(ppm)	NMOG(μg m^−3^)	eBC(μg m^−3^)^b^	POA(μg m^−3^)^b^	NO_3_(μg m^−3^)^b^	NH_4_(μg m^−3^)^b^	SO_4_(μg m^−3^)^b^	chloride(μg m^−3^)^b^	OH exposure(molec cm^−3^ h)	OA(μg m^−3^)^b^
	primary											aged	
1	0.54231 (0.00007)	537.03(0.07)	13.03(0.02)	0.8353(0.0004)	1490	104.5 (0.5)	22.9 (0.3)	17.0 (0.2)	3.80 (0.04)	0.58 (0.02)	0.153(0.005)	4.5 × 10^7^	71
2	0.33360 (0.00007)	330.40(0.07)	7.58(0.02)	0.4754(0.0004)	4320	52 (1)	17.6 (0.4)	7.6 (0.1)	1.73 (0.03)	0.27 (0.02)	0.116(0.004)	5.5 × 10^7^	117
3	0.37052 (0.00006)	366.17(0.06)	8.73(0.02)	0.6384(0.0004)	3410	113.2 (0.7)	18.8 (0.3)	14.4 (0.2)	3.15 (0.04)	0.51 (0.02)	0.240(0.006)	5.3 × 10^7^	99
4	0.59413 (0.00009)	585.73(0.09)	15.80(0.02)	1.4352(0.0005)	1860	58.1 (0.3)	18.7 (0.3)	24.7 (0.3)	6.07 (0.06)	0.84 (0.03)	0.356(0.008)	5.2 × 10^7^	114
5	0.5726 (0.0007)	568.5(0.7)	12.89(0.02)	0.945(0.001)	884	50.6 (0.4)	14.9 (0.2)	21.3 (0.2)	5.34 (0.05)	1.01 (0.04)	0.048(0.006)	4.7 × 10^7^	45

^a^Errors are given in parentheses and are ±1 s calculated from the error propagation of the sample standard deviation of the measurements. POA, OA, nitrate (NO_3_), ammonium (NH_4_), sulfate (SO_4_) and chloride are measured with an AMS, NMOGs are measured with a PTR-ToF-MS, eBC is measured with an Aethalometer and cavity ring-down spectroscopy is used to measure CO_2_, CO and CH_4_.

^b^Values are wall loss corrected.

**Table 2:** Measured and wall loss corrected SOA and fractional contribution of NMOGs to SOA after an OH exposure of (4.5–5.5) × 10^7^ molec cm^−3^ h.

species	SOA yield	experiment					average^a^
		1	2	3	4	5	
SOA_wall loss corrected_ (μg m^−3^)		71	117	99	114	45	
SOA_non-wall loss corrected_ (μg m^−3^)		33	60	51	48	27	
phenol	0.44^b^	0.34	0.13	0.20	0.28	0.36	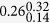
naphthalene	0.52^c^^,^^d^	0.16	0.03	0.15	0.25	0.21	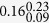
benzene	0.33^e^	0.10	0.02	0.07	0.17	0.23	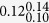
*o*-benzenediol	0.39^f^	0.05	0.14	0.11	0.03	0.05	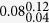
*m*-/*o*-cresol	0.36^f^	0.06	0.07	0.06	0.04	0.06	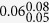
2-methoxyphenol	0.45^b^^,^^c^	0.03	0.13	0.08	0.03	0.03	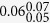
2,4-/2,6-/3,5-dimethylphenol	0.44^f^	0.02	0.04	0.03	0.01	0.02	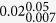
toluene	0.24^e^^,^^g^	0.02	0.009	0.02	0.03	0.03	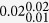
2,6-dimethoxyphenol	0.26^b^^,^^c^	0.003	0.05	0.03	0.01	0.002	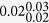
2-/3-methylfuran	0.07^h^	0.01	0.03	0.02	0.007	0.01	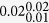
1-/2-methylnaphthalene	0.52^d^	0.01	0.003	0.01	0.02	0.02	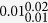
furan	0.05^h^	0.004	0.02	0.01	0.003	0.005	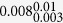
prop-2-enal	0.02^c^^,^^i^	0.005	0.006	0.006	0.004	0.005	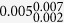
2-methylprop-2-enal/(2*E*)-2-butenal	0.03^c^^,^^i^	0.004	0.008	0.006	0.002	0.004	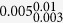
*m*-xylene	0.20^c^^,^^e^	0.005	0.004	0.004	0.005	0.007	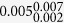
acenaphthylene	0.06^j^	0.003	0.0006	0.003	0.005	0.006	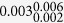
1,2-dimethylnaphthalene	0.31^d^	0.002	0.001	0.002	0.003	0.003	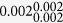
1,2-dihydroacenaphthylene	0.07^j^	0.001	BDL^l^	0.0008	0.002	0.002	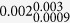
2,4-/2,5-dimethylfuran	0.32^k^	0.02	0.07	0.05	0.02	0.02	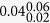
styrene	0.32^k^	0.03	0.006	0.02	0.04	0.04	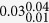
benzaldehyde	0.32^k^	0.03	0.009	0.02	0.03	0.03	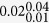
4-(2-hydroxyethyl)phenol/2-methoxy-4-methylphenol	0.32^k^	0.007	0.05	0.03	0.009	0.006	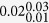
structurally assigned ≥C6 compounds	0.32^k^	0.09	0.16	0.13	0.09	0.11	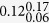
structurally unassigned ≥C6 compounds	0.32^k^	0.26	0.49	0.41	0.26	0.30	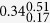

^a^Superscript values indicate upper limit and subscript values indicate lower limit based on range of upper and lower limits of the best estimate SOA yield (Table S1). For species with no literature data available and for species with only a single yield measurement (i.e., *o*-benzenediol), a yield uncertainty of ±50% is estimated.

^b^Reference 37.

^c^Reference 34.

^d^Reference 33.

^e^Reference 35.

^f^Reference 38.

^g^Reference 36.

^h^Reference 41.

^i^Reference 39.

^j^Reference 40.

^k^Average of applied yields from NMOGs with at least six carbon atoms per molecule.

^l^Below detection limit (BDL).

**Table 3:** Estimated contribution of open biomass burning emissions to global SOA.

Average biomass burned 2005–2010^a^	5731 Tg yr^−1^
Biomass burning SOA (best-estimate from top-down approaches)^b^	17 TgC yr^−1^ (0–34 TgC yr^−1^)
Biomass burning SOA (estimated using published emission factors and SOA yields)^c^	4.4 TgC yr^−1^ (0.4–20 TgC yr^−1^)

^a^Reference 57.

^b^Best estimate from reference 5. Values in parentheses indicate upper and lower limits.

^c^Estimated using emission factors from reference 56 available for 19 of the 22 NMOGs responsible for SOA formation during aging of residential wood combustion emissions. Emission factors from trash and cooking-related burning are not considered. Values in parentheses indicate upper and lower limits determined using the range of SOA yields in the literature given in Supplementary Table S1 and range of emission factors based on type of fuel combusted (i.e., lower limit corresponds to pure wiregrass burning and lowest reasonable SOA yields and upper limit corresponds to pure ponderosa pine burning and highest reasonable SOA yields).
