# Supplementary material for: Identification of significant precursor gases of secondary organic aerosols from residential wood combustion
Source: Sci Rep. 2016 Jun 17;6:27881. doi: 10.1038/srep27881 (PMC4911554; doi:10.1038/srep27881)
Supplement: Supplementary Information [file srep27881-s1.pdf]

Supplementary Information

Identification of significant precursor gases of secondary organic aerosols from residential wood combustion

Emily A. Bruns, Imad El Haddad, Jay G. Slowik, Dogushan Kilic, Felix Klein, Urs Baltensperger and André S. H. Prévôt

Methods

Beech wood (*Fagus sylvatica*, 2.9±0.3 kg; 19±2% moisture content; 3 logs, 4 pieces of kindling and 3 small ignition aids comprised of pine wood shavings, paraffin and natural resin) is combusted in a modern log burner (Avant, 2009, Attika). Emissions pass from the chimney stack through a heated line (473 K) to a diluter (473 K, DI-1000, Dekati Ltd.) and are injected into a Teflon smog chamber (~7 m^3) through a heated line (423 K). Injection into the smog chamber begins at least 15 minutes after fire ignition to avoid injecting the highly variable emissions from the starting phase into the smog chamber. Emissions are injected until atmospherically-relevant POA concentrations are reached in the chamber (Table 1). Total injection times range from 11-21 min and emissions are diluted by total factors of ~100-200.

Prior to emission injection, the smog chamber is cleaned by filling with humidified pure air and O_3, irradiating for at least 1 h with UV light (40 lights, 90-100 W, Cleo Performance, Philips) and then flushing with pure, dry air for at least 12 h. The chamber is then partially filled with humidified pure air, and background particulate and gas-phase concentrations are measured in the clean chamber. After emission injection, additional humidified pure air is added as needed to completely fill the chamber. Over the course of the experiments, the average chamber temperature (T) is 287.0±0.1 K and the average relative humidity is 55±3%.

After emission injection, primary emissions are characterized using a variety of online techniques, as described below. After characterizing the primary emissions, a single injection of d9-butanol (butanol-D9, 98%, Cambridge Isotope Laboratories) is introduced into the chamber. The decay of d9-butanol in the chamber is monitored throughout aging to determine OH exposures[1]. A continuous injection of nitrous acid in pure air (2.3-2.6 l min^{-1}, ≥99.999%, Air

Liquide)[2] into the chamber begins and the contents of the chamber are irradiated with UV light for 4-6.5 h.

Non-refractory particulate matter is characterized using a high resolution time-of-flight aerosol mass spectrometer (AMS, 1 µm lens, 600ºC vaporizer temperature, Aerodyne Research, Inc.). Particles are dried prior to analysis by AMS (Nafion membrane, Perma Pure LLC) and AMS data are analyzed in Igor Pro 6.3 (WaveMetrics, Inc.) using the SQUIRREL (version 1.53F) and PIKA (version 1.12F) analysis programs. An AMS collection efficiency of 1 is applied based on previous biomass burning studies[3-5]. Equivalent black carbon (eBC) is quantified using a 7 wavelength Aethalometer (2 l min^{-1}, AE33, Magee Scientific Company)[6]. Particle wall loss rates in the chamber are determined using the decay of eBC at the end of each experiment assuming all particles are lost equally to the walls and that condensable material partitions only to suspended particles[7]. All literature SOA yields, except the yields from Hildebrandt *et al.* [8] for toluene, are determined using the decay of an inert seed aerosol and with no consideration of partitioning of condensable vapors to the walls, as done in the current study. The average particle half-life in the chamber is 3.4±0.7 h. NMOG wall losses are inferred by monitoring NMOG concentrations prior to initiating photochemistry and by assessing the smog chamber conditions affecting loss rates[9]. Measurements of NMOGs in the chamber prior to aging are stable, indicating that the chamber walls are not a sink for NMOGs, but rather that NMOGs are in equilibrium with the chamber walls, particles and the gas phase. Zhang *et al.* [9] show that the bias created by the wall loss is inversely proportional to seed aerosol concentration and OH concentration, both of which were relatively high in the current experiments. Seed aerosol concentrations are given in Table 1 and OH concentrations during the experiments were

~1.4x10^7 molec cm^{-3}. Under these experimental conditions, NMOG wall losses are not expected to be large and thus no corrections were applied.

Gas-phase species are sampled from the chamber through a Teflon sample line heated to 323 K after exiting the temperature-controlled chamber housing. A filter (Tissuquartz, Pall Corporation) upstream of the inlets prevents particles from entering the instruments monitoring gas-phase species. NMOGs are characterized using a high resolution proton transfer reaction time-of-flight mass spectrometer (PTR-ToF-MS 8000, Ionicon Analytik G.m.b.H.), operating with hydronium ions as the reagent and with a drift tube pressure of 2.2 mbar, voltage of 543 V and temperature of 90°C. The ratio of the electric field (*E*) and the density of the buffer gas (*N*) in the drift tube, which dictates the ion drift velocity in the drift tube, is 137 Td. The PTR-ToF-MS transmission function is determined using six NMOGs in a gas standard (methanol, acetaldehyde, propan-2-one, toluene, *p*-xylene, 1,3,5-trimethylbenzene; Carbagas). PTR-ToF-MS data are analyzed in Igor Pro 6.3 (WaveMetrics, Inc.) using the Tofware analysis platform (version 2.4.5, Tofwerk). Mass spectral data from *m/z* 33 to *m/z* 130 are fit, as well as the ^{18}O isotope of the reagent ion. Peak widths and possible molecular formulas increase with increasing *m/z*, making accurate peak assignments difficult in the higher *m/z* range. However, signal above *m/z* 130 corresponding to compounds previously identified during residential wood combustion are fit[10-16]. Isotopic contributions are constrained during peak fitting and accounted for when determining parent peak concentrations. The minimum detection limit is taken as three standard deviations above the background, where the standard deviation is determined from the background measurements of each ion in the chamber prior to emission injection. There is a small continuous dilution in the chamber during aging due to the constant nitrous acid injection and NMOG time traces are corrected for this dilution using CO as an inert tracer.

Assigning structures to ions detected using the PTR-ToF-MS is critical for understanding the conversion of NMOGs to SOA during aging. Structural assignments are guided by previously identified compounds emitted during residential wood combustion[10-16]. The contribution of an individual NMOG to the measured SOA is determined using the temporal evolution of the compound during aging and published SOA yields[8,17-24], which are available for 18 of the 59 identified compounds. SOA yields can depend on experimental conditions, including the ratio of NO_x to NMOG, presence of seed aerosol and total organic aerosol mass[8,18,19,22,25]. When a range of yields are available, the average of literature values from experiments most similar to the current study in terms of seed aerosol and NO_x/NMOG is applied. The average NO_x/NMOG for the set of experiments is ~150 ppb $ppmC^{-1}$ and individual values ranges from ~35-350 ppb $ppmC^{-1}$. In addition to the 18 species with SOA yields in the literature, isomers of 2,4-/2,5-dimethylfuran, styrene, benzaldehyde and isomers of 4-(2-hydroxyethyl)phenol/2-methoxy-4-methylphenol are identified as likely contributors to SOA due to relatively high concentrations and structural similarities to the compounds for which SOA yields are known. The SOA yields for these four compounds are taken as the average of the published SOA yields for the NMOGs with at least 6 carbon atoms per molecule ($\geq C_6$). This estimated SOA yield is also applied to the sum of compounds with lower relative concentrations and at least six carbon atoms per molecule (structurally assigned $\geq C_6$ compounds), as well as signal above *m/z* 130 which is not fit, but is expected to be due to compounds with at least six carbon atoms per molecule and could contribute to SOA formation (structurally unassigned $\geq C_6$ compounds).

Uncertainties arise from the inability to resolve isomers using the PTR-ToF-MS. For example, SOA yields are available for both 2-methylprop-2-enal and (*2E*)-2-butenal and the applied yield is taken as an average of the values from the two isomers. SOA yields are relatively low for both

of these compounds and the impact on results is negligible. In cases of possible isomeric contributions where a SOA yield is available for only one isomer, the approach assumes that either 1) the isomers have the same SOA yield or 2) the signal is entirely due to the compound with the known SOA yield. The possibility of isomers is not expected to influence the results considerably, however, as the majority of compounds are not suspected to have significant isomer contributions (Table S2) and the compounds that contribute the most to the SOA have no previously detected isomers in residential wood combustion emissions.

The reaction rate constant of each species with the reagent in the drift tube is needed to convert the raw PTR-ToF-MS signal to concentration. The relatively limited availability of applicable measured reaction rate constants precludes assignment of a reaction rate constant to each ion. When available, individual reaction rate constants are applied[26] and a default reaction rate constant of 2×10^{-9} cm^3 s^{-1} is applied to all other ions. In cases where ions could correspond to several isomers, the reaction rate constant is taken as the average of available values. Reaction rate constants varying from the default for species of interest for SOA formation are presented in Table S3.

Monoterpene emissions

Monoterpenes have been previously measured in residential wood combustion[10,12], but are below the detection limit in all experiments, which is likely largely due to the type of wood burned. Previously reported emission factors (mg kg^{-1} should be read as mg emitted species per kg combusted fuel) for wood stove burning of beech wood are below the detection limit for 3-carene and limonene and very low for α-pinene (0.506 mg kg^{-1})[12]. α-Pinene emissions of this magnitude would contribute less than 0.2% to SOA in the current study. The emission factors

for other compounds reported by Evtyugina *et al.* [12] for wood stove burning of beech wood, except naphthalene which was below the detection limit, are within a factor of ~1-3 of those in the current study, which is reasonable considering the large impact of burn parameters on emissions[27]. Although it is reasonable that monoterpenes are not detected based on previous findings[12], these species can be emitted in much larger quantities during the burning of other wood types, particularly softwoods[10] which can emit over ten times more terpenes than during comparable burning of hardwoods, and should be considered in models in addition to the 22 individually identified NMOGs.

References

1. Barmet, P., *et al.* OH clock determination by proton transfer reaction mass spectrometry at an environmental chamber. *Atmos Meas Tech* **5**, 647-656 (2012).

2. Taira, M. & Kanda, Y. Continuous generation system for low-concentration gaseous nitrous-acid. *Anal Chem* **62**, 630-633 (1990).

3. Heringa, M. F., *et al.* Investigations of primary and secondary particulate matter of different wood combustion appliances with a high-resolution time-of-flight aerosol mass spectrometer. *Atmos Chem Phys* **11**, 5945-5957 (2011).

4. Eriksson, A. C., *et al.* Particulate PAH emissions from residential biomass combustion: time-resolved analysis with aerosol mass spectrometry. *Environ Sci Technol* **48**, 7143-7150 (2014).

5. Hennigan, C. J., *et al.* Chemical and physical transformations of organic aerosol from the photo-oxidation of open biomass burning emissions in an environmental chamber. *Atmos Chem Phys* **11**, 7669-7686 (2011).

6. Drinovec, L., *et al.* The "dual-spot" Aethalometer: an improved measurement of aerosol black carbon with real-time loading compensation. *Atmos Meas Tech* **8**, 1965-1979 (2015).

7. Weitkamp, E. A., Sage, A. M., Pierce, J. R., Donahue, N. M. & Robinson, A. L. Organic aerosol formation from photochemical oxidation of diesel exhaust in a smog chamber. *Environ Sci Technol* **41**, 6969-6975 (2007).

8. Hildebrandt, L., Donahue, N. M. & Pandis, S. N. High formation of secondary organic aerosol from the photo-oxidation of toluene. *Atmos Chem Phys* **9**, 2973-2986 (2009).

9. Zhang, X., *et al.* Influence of vapor wall loss in laboratory chambers on yields of secondary organic aerosol. *Proc Natl Acad Sci USA* **111**, 5802–5807 (2014).

10. McDonald, J. D., *et al.* Fine particle and gaseous emission rates from residential wood combustion. *Environ Sci Technol* **34**, 2080-2091 (2000).

11. Schauer, J. J., Kleeman, M. J., Cass, G. R. & Simoneit, B. R. T. Measurement of emissions from air pollution sources. 3. C_1-C_{29} organic compounds from fireplace combustion of wood. *Environ Sci Technol* **35**, 1716-1728 (2001).

12. Evtyugina, M., *et al.* VOC emissions from residential combustion of Southern and mid-European woods. *Atmos Environ* **83**, 90-98 (2014).

13. Hedberg, E., *et al.* Chemical and physical characterization of emissions from birch wood combustion in a wood stove. *Atmos Environ* **36**, 4823-4837 (2002).

14. Reda, A. A., *et al.* Analysis of gas-phase carbonyl compounds in emissions from modern wood combustion appliances: influence of wood type and combustion appliance. *Energ Fuel* **29**, 3897-3907 (2015).

15. Pettersson, E., Boman, C., Westerholm, R., Boström, D. & Nordin, A. Stove performance and emission characteristics in residential wood log and pellet combustion, part 2: wood stove. *Energ Fuel* **25**, 315-323 (2011).

16. Jordan, T. B. & Seen, A. J. Effect of airflow setting on the organic composition of woodheater emissions. *Environ Sci Technol* **39**, 3601-3610 (2005).

17. Chan, A. W. H., *et al.* Role of aldehyde chemistry and NO_x concentrations in secondary organic aerosol formation. *Atmos Chem Phys* **10**, 7169-7188 (2010).

18. Chhabra, P. S., *et al.* Elemental composition and oxidation of chamber organic aerosol. *Atmos Chem Phys* **11**, 8827-8845 (2011).

19. Ng, N. L., *et al.* Secondary organic aerosol formation from *m*-xylene, toluene, and benzene. *Atmos Chem Phys* **7**, 3909-3922 (2007).

20. Yee, L. D., *et al.* Secondary organic aerosol formation from biomass burning intermediates: phenol and methoxyphenols. *Atmos Chem Phys* **13**, 8019-8043 (2013).

21. Nakao, S., Clark, C., Tang, P., Sato, K. & Cocker III, D. Secondary organic aerosol formation from phenolic compounds in the absence of NO_x. *Atmos Chem Phys* **11**, 10649-10660 (2011).

22. Chan, A. W. H., *et al.* Secondary organic aerosol formation from photooxidation of naphthalene and alkylnaphthalenes: implications for oxidation of intermediate volatility organic compounds (IVOCs). *Atmos Chem Phys* **9**, 3049-3060 (2009).

23. Shakya, K. M. & Griffin, R. J. Secondary organic aerosol from photooxidation of polycyclic aromatic hydrocarbons. *Environ Sci Technol* **44**, 8134-8139 (2010).

24. Gómez Alvarez, E., Borrás, E., Viidanoja, J. & Hjorth, J. Unsaturated dicarbonyl products from the OH-initiated photo-oxidation of furan, 2-methylfuran and 3-methylfuran. *Atmos Environ* **43**, 1603-1612 (2009).

25. Odum, J. R., *et al.* Gas/particle partitioning and secondary organic aerosol yields. *Environ Sci Technol* **30**, 2580-2585 (1996).

26. Cappellin, L., *et al.* On quantitative determination of volatile organic compound concentrations using proton transfer reaction time-of-flight mass spectrometry. *Environ Sci Technol* **46**, 2283-2290 (2012).

27. Bruns, E. A., *et al.* Characterization of primary and secondary wood combustion products generated under different burner loads. *Atmos Chem Phys* **15**, 2825-2841 (2015).

28. Canagaratna, M. R., *et al.* Elemental ratio measurements of organic compounds using aerosol mass spectrometry: characterization, improved calibration, and implications. *Atmos Chem Phys* **15**, 253-272 (2015).

29. Aiken, A. C., *et al.* O/C and OM/OC ratios of primary, secondary, and ambient organic aerosols with high-resolution time-of-flight aerosol mass spectrometry. *Environ Sci Technol* **42**, 4478-4485 (2008).

30. Kautzman, K. E., *et al.* Chemical composition of gas- and aerosol-phase products from the photooxidation of naphthalene. *J Phys Chem A* **114**, 913-934 (2010).

31. Ng, N. L., *et al.* Changes in organic aerosol composition with aging inferred from aerosol mass spectra. *Atmos Chem Phys* **11**, 6465-6474 (2011).

32. Ortega, A. M., *et al.* Secondary organic aerosol formation and primary organic aerosol oxidation from biomass-burning smoke in a flow reactor during FLAME-3. *Atmos Chem Phys* **13**, 11551-11571 (2013).

33. Cubison, M. J., *et al.* Effects of aging on organic aerosol from open biomass burning smoke in aircraft and laboratory studies. *Atmos Chem Phys* **11**, 12049-12064 (2011).

34. Canonaco, F., Slowik, J. G., Baltensperger, U. & Prévôt, A. S. H. Seasonal differences in oxygenated organic aerosol composition: implications for emissions sources and factor analysis. *Atmos Chem Phys* **15**, 6993-7002 (2015).

Table S1. Literature yield data and application of SOA yields

	literature data				application of yield data	
**compound**	**yield reference[a]**	**chamber T (K)**	**seed**	**NO_x/NMOG (ppb ppmC^{-1})[b]**	**range of yields used to determine applied yield[c]**	**applied yield**
phenol	20	293-299	yes	≈0-100; ≈5600-8300 (all)	0.24-0.54	0.44
naphthalene	22<br>18	299<br>293-295	yes<br>yes	0; ≈1800-4500 (<2000)<br>0; ≈3200 (all)	0.30-0.74	0.52
benzene	19	297-298	yes	0; ≈700 (all)	0.28-0.37	0.33
o-benzenediol	21	300	no	0 (all)	n/a	0.39
m-/*o*-cresol	21	300	no	0 (all)	0.27-0.49	0.36
2-methoxyphenol	18<br>20	293-295<br>293-299	yes<br>yes	0; ≈14000 (all)<br>≈0; ≈2100-8300 (all)	0.34-0.53	0.45
2,4-/2,6-/3,5-dimethylphenol	21	300	no	0 (all)	0.13-0.9	0.44
toluene	19<br>8	296-299<br>284-305	yes<br>yes	0; ≈200-250; ≈1700-4500 (≈200-250)<br>0; ≈100-300 (≈100-300)	0.12-0.44	0.24
2,6-dimethoxyphenol	18<br>20	293-295<br>293-299	yes<br>yes	0 (all)<br>≈0-100; ≈800-2600 (<1000)	0.20-0.37	0.26
2-/3-methylfuran	24	n/a (ambient)	no	≈20-30	0.055-0.085	0.07
1-/2-methylnaphthalene	22	299	yes	0; ≈2000-6700 (<4000)	0.38-0.71	0.52
furan	24	n/a (ambient)	no	≈20-30	0.019-0.072	0.05
prop-2-enal	17<br>18	293-295<br>293-295	yes<br>yes	≈300-1700 (<600)<br>≈500 (all)	0.006-0.023	0.02
2-methylprop-2-enal/ (2*E*)-2-butenal	17<br>18	293-295<br>293-295	yes<br>yes	≈500-9700 (<1000)<br>≈600-1500 (<1000)	0.013-0.052	0.03
m-xylene	19<br>18	297-298<br>293-295	yes<br>yes	0, ≈200-4200 (≈200)<br>0; ≈600 (≈600)	0.06-0.26	0.20
acenaphthylene	23	294-297	no	≈500-1600 (all)	0.03-0.11	0.06
1,2-dimethylnaphthalene	22	299	yes	≈1100-9000 (all)	0.30-0.31	0.31
1,2-dihydroacenaphthylene	23	294-297	no	≈300-2400 (all)	0.04-0.13	0.07
2,4-/2,5-dimethylfuran	n/a	n/a	n/a	n/a	n/a	0.32[d]
styrene	n/a	n/a	n/a	n/a	n/a	0.32[d]
benzaldehyde	n/a	n/a	n/a	n/a	n/a	0.32[d]
4-(2-hydroxyethyl)phenol/2-methoxy-4-methylphenol	n/a	n/a	n/a	n/a	n/a	0.32[d]
structurally assigned ≥C_6 compounds	n/a	n/a	n/a	n/a	n/a	0.32[d]
structurally unassigned ≥C_6 compounds	n/a	n/a	n/a	n/a	n/a	0.32[d]

[a]Yields obtained without seed aerosol are not included if yields obtained with seed aerosol are available. Yields measured under vastly different NO_x/NMOG ratios are not included if yields obtained under NO_x/NMOG ratios more similar to the current study are available.
[b]Information in parentheses indicates conditions where yield data are used to determine applied yield.
[c]Yield range is used to determine upper and lower best estimate contributions to observed SOA.
[d]Yield determined from average of applied yields from NMOGs in the table with at least 6 carbon atoms per molecule.

Table S2. Structural assignment of ions relevant to SOA formation detected using PTR-ToF-MS including isomers previously detected in residential wood combustion emissions

ion detected by PTR-MS	nominal *m/z*	structural assignment(s)
compounds with published SOA yields[a]		
$[C_3H_4O+H]^+$	57	prop-2-enal
$[C_4H_4O+H]^+$	69	furan
$[C_4H_6O+H]^+$	71	2-methylprop-2-enal/(2*E*)-2-butenal (3-butene-2-one)
$[C_6H_6+H]^+$	79	benzene
$[C_7H_8+H]^+$	93	toluene
$[C_5H_6O+H]^+$	83	2-/3-methylfuran
$[C_6H_6O+H]^+$	95	phenol
$[C_8H_{10}+H]^+$	107	*m*-xylene (*o*-/*p*-xylene; ethylbenzene)
$[C_7H_8O+H]^+$	109	*m*-/*o*-cresol (*p*-cresol)
$[C_6H_6O_2+H]^+$	111	*o*-benzenediol (*m*-/*p*-benzenediol; 2-methylfuraldehyde)
$[C_8H_{10}O+H]^+$	123	2,4-/2,6-/3,5-dimethylphenol
$[C_7H_8O_2+H]^+$	125	2-methoxyphenol (methylbenzenediol isomers)
$[C_{10}H_8+H]^+$	129	naphthalene
$[C_{11}H_{10}+H]^+$	143	1-/2-methylnaphthalene
$[C_{12}H_8+H]^+$	153	acenaphthylene
$[C_8H_{10}O_3+H]^+$	155	2,6-dimethoxyphenol
$[C_{12}H_{10}+H]^+$	155	1,2-dihydroacenaphthylene (1,1'-biphenyl)
$[C_{12}H_{12}+H]^+$	157	1,2-dimethylnaphthalene
compounds with estimated SOA yields		
$[C_6H_8O+H]^+$	97	2,4-/2,5-dimethylfuran
$[C_8H_8+H]^+$	105	styrene
$[C_7H_6O+H]^+$	107	benzaldehyde
$[C_8H_{10}O_2+H]^+$	139	4-(2-hydroxyethyl)phenol/2-methoxy-4-methylphenol
$[C_9H_6O_2+H]^+$	147	2,3-dihydroinden-1-one
$[C_8H_8O_3+H]^+$	153	4-hydroxy-3-methoxybenzaldehyde
$[C_9H_{12}O_2+H]^+$	153	4-ethyl-2-methoxyphenol/1,2-dimethoxy-4-methylbenzene
$[C_{10}H_{12}O_2+H]^+$	165	2-methoxy-4-prop-2-enylphenol/2-methoxy-4-[(*Z*)-prop-1-enyl]phenol/ 2-methoxy-4-[(*E*)-prop-1-enyl]phenol
$[C_9H_{10}O_3+H]^+$	167	1-(4-hydroxy-3-methoxyphenyl)ethanone/2,5-dimethylbenzaldehyde/3,4-dimethoxybenzaldehyde
$[C_{13}H_{10}+H]^+$	167	fluorene
$[C_{10}H_{14}O_2+H]^+$	167	2-methoxy-4-propylphenol
$[C_9H_{12}O_3 +H]^+$	169	2,6-dimethoxy-4-methylphenol
$[C_{14}H_{10}+H]^+$	179	phenanthrene/anthracene
$[C_{13}H_8O +H]^+$	181	fluoren-9-one/phenalen-1-one
$[C_{10}H_{12}O_3+H]^+$	181	1-(4-hydroxy-3-methoxyphenyl)propan-2-one
$[C_9H_{10}O_4+H]^+$	183	3,4-dimethoxybenzoic acid/4-hydroxy-3,5-dimethoxybenzaldehyde
$[C_{10}H_{14}O_3+H]^+$	183	4-ethyl-2,6-dimethoxyphenol
$[C_{15}H_{12}+H]^+$	193	3-methylphenanthrene/2-methylphenanthrene/1-methylphenanthrene/9-methylphenanthrene/2-methylanthracene
$[C_{11}H_{14}O_3+H]^+$	195	1,3-dimethoxy-2-prop-2-enoxybenzene /2,6-dimethoxy-4-[(*Z*)-prop-1-enyl]phenol
$[C_{16}H_{10}+H]^+$	203	fluoranthene/pyrene/acephenanthrylene

[a]For compounds with published SOA yields, isomers for which SOA yields are not known are given in parentheses.

Table S3. PTR-ToF-MS reagent ion reaction rates[a,b]

ion	species	reaction rate ($\times 10^{-9}$ cm^3 s^{-1})
$[C_3H_4O+H]^+$	prop-2-enal	3.43
$[C_4H_4O+H]^+$	furan	1.69
$[C_4H_6O+H]^+$	2-methylprop-2-enal/(2*E*)-2-butenal (3-butene-2-one)	3.16
$[C_6H_6+H]^+$	benzene	1.93
$[C_7H_8+H]^+$	toluene	2.08
$[C_6H_6O+H]^+$	phenol	2.13
$[C_8H_8+H]^+$	styrene	2.27
$[C_7H_6O+H]^+$	benzaldehyde	3.63
$[C_8H_{10}+H]^+$	*m*-xylene (*o*-/*p*-xylene, ethylbenzene)	2.26
$[C_7H_8O+H]^+$	*m*-/*o*-cresol	2.27
$[C_{10}H_8+H]^+$	naphthalene	2.45
$[C_{11}H_{10}+H]^+$	1-/2-methylnaphthalene	2.71
$[C_{12}H_8+H]^+$	acenaphthylene	2.86
$[C_{12}H_{10}+H]^+$	1,2-dihydroacenaphthylene (1,1'-biphenyl)	2.81
$[C_{13}H_{10}+H]^+$	fluorene	2.88
$[C_{14}H_{10}+H]^+$	phenanthrene/anthracene	3.09
$[C_{16}H_{10}+H]^+$	fluoranthene/pyrene/acephenanthrylene	3.37

[a]Reaction rates from reference 25 at a drift tube temperature of 90ºC and an *E*/*N* of 140 Td. A reaction rate of 2×10^{-9} cm^3 s^{-1} is applied to all other ions.

[b]In the cases of possible isomers previously identified in residential wood combustion emissions, given in parentheses, an average of the available rate constants is applied.

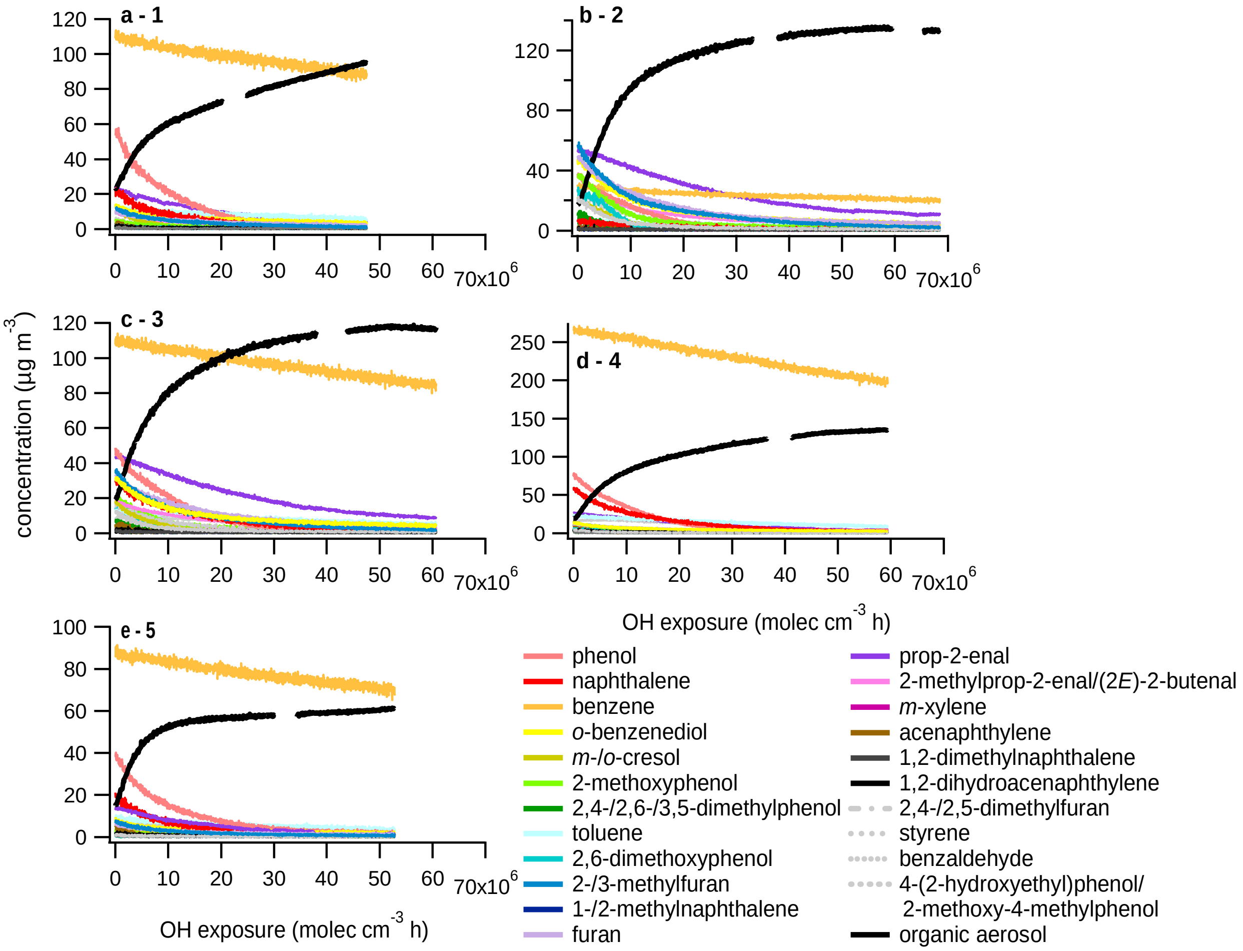

Figure S1. Evolution of 22 individual NMOGs and organic aerosol with aging in each experiment (1-5, a-e). The black time traces correspond to wall loss corrected organic aerosol, the other solid traces correspond to 18 individual NMOGs for which SOA yields are reported in the literature and the dashed gray traces correspond to the 4 NMOGs with an estimated SOA yield.

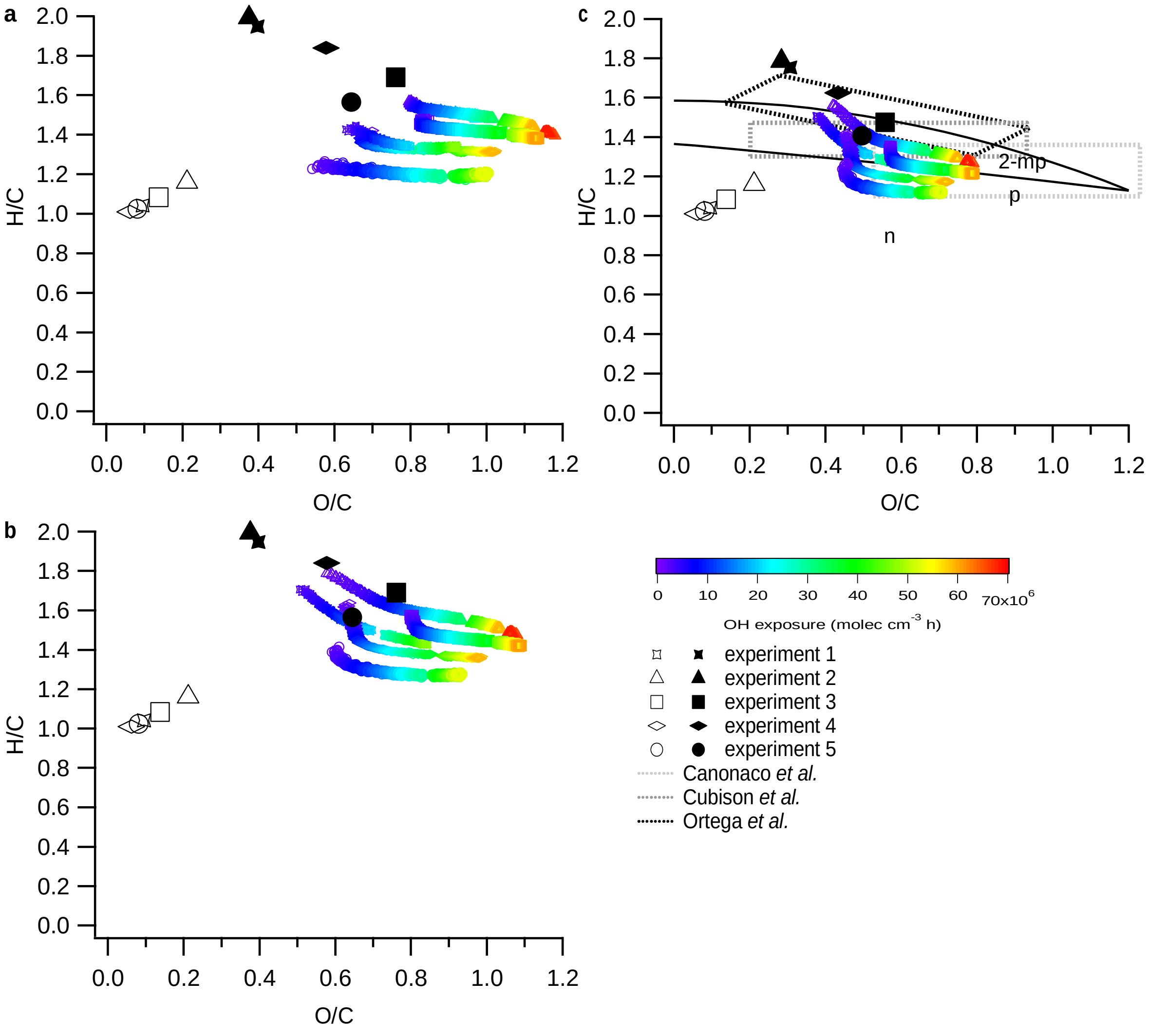

Figure S2. Elemental composition of SOA, POA and mass concentration-weighted average of 22 primary NMOGs. Colored traces correspond to elemental ratios as a function of OH exposure of (a) SOA and (b) total OA determined using Canagaratna *et al.* [28] parameterizations, and (c) total OA determined using Aiken *et al.* [29] parameterizations. Only data points corresponding to SOA/POA≥1 in the chamber are shown. Open black data points correspond to NMOGs and solid black data points correspond to POA determined using the same parameterizations as the colored traces. (c) Lettered data points correspond to literature values using the Aiken parameterization from SOA formed during aging of individual precursors[18,30], solid black lines correspond to the region encompassing typical ambient experiments[31] and dashed gray boxes encompass measurements of laboratory open biomass burning SOA[32] and ambient OA measurements impacted by open biomass burning[33] and residential burning[34]. Literature burning data are transformed to H/C and O/C using the parameterizations of Ng *et al.* [31] when needed.

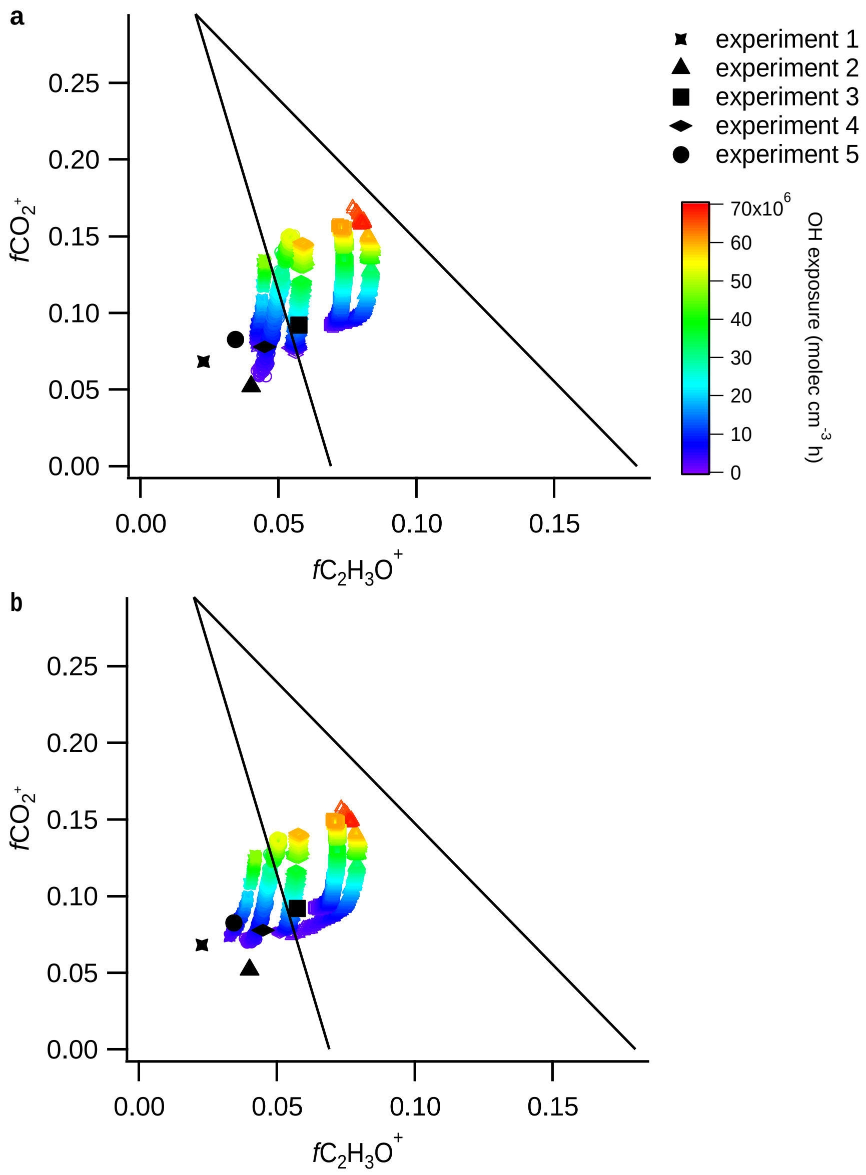

Figure S3. AMS fraction of CO_2^+ and $C_2H_3O^+$ to (a) SOA and (b) total OA. Colored traces correspond to fractions as a function of OH exposure when SOA/POA in the chamber is ≥1. Solid black data points correspond to POA and solid black lines correspond to the region encompassing typical ambient experiments[31]. Legend in (a) also applied to (b).
